# Supplementary material for: Conditional chemoconnectomics (cCCTomics) as a strategy for efficient and conditional targeting of chemical transmission
Source: eLife. 2024 Apr 30;12:RP91927. doi: 10.7554/eLife.91927 (PMC11060718; doi:10.7554/eLife.91927)
Supplement: Figure 3—source data 3. [file elife-91927-fig3-data3.zip › Figure 3 source data3 part 2.zip!/Figure 3-Source data 3-part2/1053f3-1-CRISPResso_Report_t6djfp6u.zip!/CRISPResso_on_1053f3_2/3b.Insertion_deletion_substitutions_size_hist.pdf]

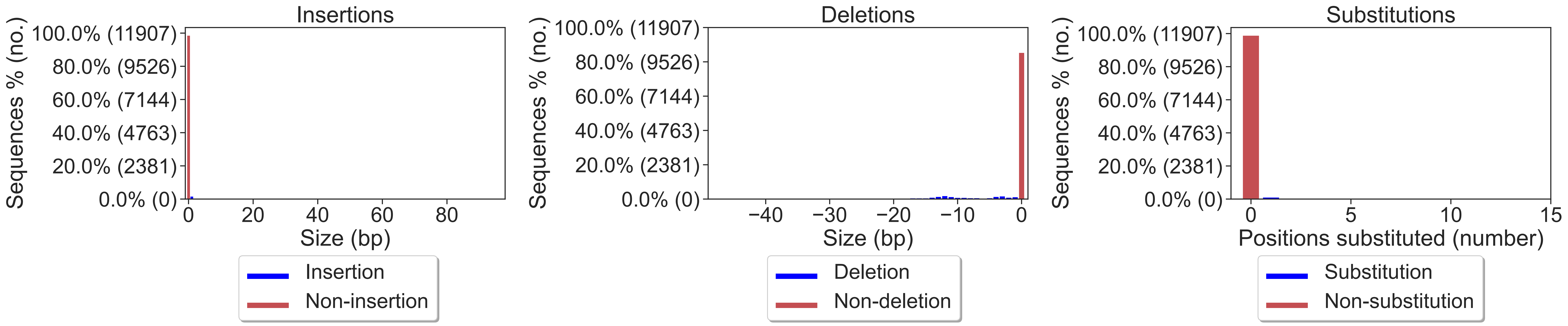

Insertions
Deletions
Substitutions
Sequences % (no.)
100.0% (11907)
80.0% (9526)
60.0% (7144)
40.0% (4763)
20.0% (2381)
0.0% (0)
0
20
40
60
80
−40
−30
−20
−10
5
10
15
Size (bp)
Positions substituted (number)
Insertion
Non-insertion
Deletion
Non-deletion
Substitution
Non-substitution
